# Feminism, gender medicine and beyond: a feminist analysis of "gender medicine"

**DOI:** 10.1186/s12939-021-01511-5

**Published:** 2021-08-03

**Authors:** Ayelet Shai, Shahar Koffler, Yael Hashiloni-Dolev

**Affiliations:** 1 Oncology Department, Gailee Medical Center, 89 Meona rd, Nahariya, Israel; 2 Azrieli Faculty of Medicine, Bar-Ilan University, Safed, Israel; 3 Pediatrics Department, Samson Assuta Ashdod University Hospital, Ashdod, Israel; 4 Sociology and Anthropology Department, Ben-Gurion University of the Negev, Ben- Gurion Blvd 1, Be’er-Sheva, Israel

**Keywords:** Gender medicine, Feminism, women’s health movement, Sexual abuse, Sex/gender medicine, women’s health

## Abstract

The feminist women’s health movement empowered women’s knowledge regarding their health and battled against paternalistic and oppressive practices within healthcare systems. Gender Medicine (GM) is a new discipline that studies the effect of sex/gender on general health. The international society for gender medicine (IGM) was embraced by the FDA and granted funds by the European Union to formulate policies for medical practice and research.

We conducted a review of IGM publications and policy statements in scientific journals and popular media. We found that while biological differences between men and women are emphasized, the impact of society on women is under- represented. The effect of gender-related violence, race, ethnic conflicts, poverty, immigration and discrimination on women’s health is seldom recognized. Contrary to feminist practice, GM is practiced by physicians and scientists, neglecting voices of other disciplines and of women themselves.

In this article we show that while GM may promote some aspects of women’s health, at the same time it reaffirms conservative positions on sex and gender that can serve to justify discrimination and disregard the impact of society on women’s lives and health. An alternative approach, that integrates feminist thinking and practices into medical science, practice and policies is likely to result in a deep and beneficiary change in women’s health worldwide.

## Introduction

The women’s health movement, which emerged during the 1960s and 1970s along with the second wave of feminism, recognized the female body as the vessel that mediates male dominance. Feminists demanded improved healthcare and the elimination of sexism in healthcare systems. Activists fought to empower women’s knowledge, gain control over reproductive rights, and reclaim power from the paternalistic medical community [1]. They likewise battled against the oppression of women, manifest in the denial of access to abortions and contraceptives, prostitution, sexual violence, pornography, and beauty industry standards. Later, feminists criticized the medicalization and commercialization of reproduction and labor and the exploitation of underprivileged women in the reproductive industry [2].

Feminist thinkers coined the term “gender” to differentiate between biological and social aspects of being male or female and to emphasize the role of culture and society in the construction of human sexuality [3]. Later thought problematized the biological category of sex itself, pointing to it as a social construct no less than gender [4]. Moreover, recent scientific evidence reveals that it is impossible to separate sex and gender [5, 6], and that the dichotomy of two sexes is ignoring a more complex biological and social reality [7].

The new discipline of gender medicine (GM) aspires to examine the influence of gender on general medical issues. It argues that modern medical knowledge is based on observations and trials conducted mainly on men and that this wrong should be righted to achieve medical knowledge better suited to women [8]. The International Society for Gender Medicine (IGM) was founded in 2006 and was embraced by the European Union and the FDA [9]. It is consulted by institutions such as the Israeli parliament [10] and professional societies, for example the European Federation of Internal Medicine [11]. The IGM was granted financial resources from the European Union to promote its cause [12] and holds international conferences. Recently, medical schools introduced GM into their curricula. Since many consider the IGM to be representative of women’s health interests, it is vital to assess its views and actions and their implications for women [13]. Moreover, the recognition of GM as a discipline and its endorsement by the professional milieu is an opportunity to assess the attitude of the bio-medical world to feminist thinking and criticism. Thus, our goal was to analyze GM from a feminist perspective.

## Methods

For this purpose, we reviewed scientific publications by past and present officials of IGM and of the Israeli Society for Gender and Sex Conscious Medicine (ISGSCM), listed on their websites, as well as their public appearances and press interviews. The scientific literature review included 27 articles concerning sex/gender-related issues published from 2010 until May 2020 in journals with an impact factor of 4 or more or a rank of 40 or less. In addition, we reviewed the report of the European Gender Medicine Network (EUGENMED) [12], an extensive project held between 2013 and 2015, that aimed to summarize the scientific data on gender and medicine and formulate recommendation for future policies. The popular media literature review included 24 relevant interviews and articles that were retrieved by searching the internet for entries containing the names of the IGM and the ISGSCM officials and reviewing their content. Interviews and articles in English and Hebrew, containing discussions on sex/gender and medicine, were included. Popular media publications were included in the analysis because GM has an explicit political agenda which it aims to promote also in popular venues.

Our study builds on the Foucauldian analysis of knowledge looking into the relationship between discourse and power, through the lens of the discourse of professional disciplines, in order to study the boundaries of thought used in a given time and discipline [14]. Thus, we analyzed the studied texts through the lens of power/knowledge relationships, ideology, and inequality. We formed two integrated files, one consisting of medical publications, the second of texts from the media. The texts were analyzed using a qualitative analysis method. Main themes concerning sex/gender and medicine were extracted from the texts inductively [15]. In addition, in dialog with themes in the feminist literature, we searched for what is missing in the discussion, in a deductive manner. The first two authors of the study who are MD’s read and discussed in several rounds the first medical file, identifying themes, and matching them with the relevant literature. The second file was analyzed by the third author, who is a social scientist. As a second step agreements were reached between all authors to prevent the potential bias of a single researcher and using inter-rater reliability to increase the validity of the results.

We hereby critically assess these publications in the context of current feminist thinking, noting both the topics discussed and those that were overlooked, or only seldom mentioned. After presenting our findings we discuss their implications.

### Analysis of GM works

The scientific articles we reviewed focus on several subjects: Seventeen articles focused on the association between sex/gender, risk of disease and response to therapy, mainly in the field of cardiovascular diseases and related disorders [16–32]. Two articles studied the influence of sex/gender on treatment decisions and care plans [33, 34]; 5 articles focused on associations between sex/gender and the human brain, cognition, and mood [25, 35–38] ; 2 dealt with the effect of sex/gender on working conditions and promotion in healthcare [39, 40], 2 focused on sex/gender in medical education [41, 42] and 1 focused on sex in preclinical research [43]. The articles we reviewed are summarized in Table 1. The EUGENMED project summary reported on 5 working fields: 1. Sex/gender, risk of disease and treatment outcomes in cardiovascular medicine, pulmonary medicine, diabetes mellitus and psychiatry (depression). 2. Sex/gender and public health, focusing on risk factors for non-communicable diseases 3. Sex/gender in basic research. 4. Sex/gender in medical education. 5. Sex/gender and pharmacology, clinical trials and pharmaceutical regulation [12]. Each summary of a working field contained a detailed review of scientific literature and advocacy for future actions.

**Table 1:** Summary of the GM scientific publications that were reviewed

Reference number	Article title	General subject	Main findings
[16]	Sex-specific analysis of hemodialysis prevalence, practices, and mortality over time: The Austrian Dialysis Registry from 1965 to 2014	Sex/gender and health outcomes: renal failure	• Women with renal failure were less likely to undergo hemodialysis. • Lower rates of kidney transplant in women. • Higher mortality for men with ESRD compared to women. • Higher mortality due to dialysis access in women.
[17]	Gender in cardiovascular diseases: Impact on clinical manifestations, management, and outcomes	Sex/gender and health outcomes: Cardiovascular diseases and related disorders	• Coronary angiography should not be used as a diagnostic procedure for low to intermediate risk women. • Consideration of gender may be helpful to decide on most efficient treatment strategies in valvular diseases- the use of TAVI or conventional surgery. • Resynchronization therapy is less frequently used in women but has a greater benefit in women then in men. • Implanted defibrillators may be more efficient in men. • Gendered approaches may lead to a more specific and effective use of resource
[18]	Effects of age, gender, and body mass index on efficacy and hypoglycemia outcomes across treat-to-target trials with insulin glargine 100 U/mL added to oral anti-diabetes agents in type 2 diabetes	Sex/gender and health outcomes: diabetes mellitus.	• Female patients with type 2 diabetes treated with Glargine 100 U/mL and Metformin +/- Sulfonylurea are less likely to achieve glycemic targets relatively to male patients and may require more clinical attention.
[19]	Impact of Diabetes Mellitus on Ischemic Events in Men and Women After Percutaneous Coronary Intervention	Sex/gender and health outcomes: Cardiovascular diseases and related disorders	• Diabetes mellitus and female gender effect post-PCI risk of ischemic events. • Risk is equivalent for nondiabetic women and diabetic men.
[20]	Sex-specific-differences in cardiometabolic risk in type 1 diabetes: A cross-sectional study	Sex/gender and health outcomes: Diabetes mellitus.	• There are sex differences in lipids and weight in patients with T1DM. • Glycemic control and frequency of diabetic complications were comparable between the sexes. • Adherence to treatment guidelines was lower in women than in men.
[21]	Similarities in trabecular hypertrophy with site-specific differences in cortical morphology between men and women with type 2 diabetes mellitus	Sex/gender and health outcomes: Diabetes mellitus.	• Skeletal hypertrophy associated with T2DM is present in men and women but appears attenuated at the tibial cortex in men.
[22]	Sex and gender differences in risk, pathophysiology, and complications of type 2 diabetes mellitus	Sex/gender and health outcomes: Diabetes mellitus.	• Sex influences on vulnerability to cardiometabolic risk factors, manifestation, clinical picture, and management of T2DM. • Severity of injury differs in a sex-specific way especially regarding cardiovascular and renal disease. • Psychosocial factors impact development and progression of diabetes and coping in a gender-dimorphic way. • Offsprings of hyperglycemic parents may be at greater risk for DM. • Modern personalized treatment has to consider gender differences.
[23]	Gender, aging and longevity in humans: An update of an intriguing/neglected scenario paving the way to a gender-specific medicine	Sex/gender and health outcomes	• Gender-specific medicine approach should be established and systematically pursued in studies on healthy aging, longevity, and age-related diseases. • Gender differences have a high impact on health and diseases.
[24]	Gender medicine: A task for the third millennium	Sex/gender and health outcomes	• There are gender differences in risk factors, clinical manifestations, and treatment efficacy in CVD. • Gender differences in incidence, aggressiveness, and prognosis in variety of cancers. • Gender differences in epidemiology and progress of certain liver diseases. • Osteoporosis is underestimated in women, but patients’ and physicians’ awareness is even lower for male osteoporosis.
[25]	Sex in basic research: Concepts in the cardiovascular field	1.Sex/gender and health outcomes. 2. The human brain, cognition, and mood	• Reviews basic research work that suggests possible biological mechanisms mediating sex differences in health and in the brain. • Advocates for considering sex and in basic research and suggests the means to do so. • Advocates for encouraging scientists to study sex differences in basic research by grants and publication policies.
[26]	Sex differences in arterial wave reflection and the role of exogenous and endogenous sex hormones: results of the Berlin Aging Study II	Sex/gender and health outcomes: Cardiovascular diseases and related disorders	• Mean augmentation index (Aix) was higher in women than in men. • Oral contraceptive (OCP) use was associated with a higher Aix. Low endogenous estradiol was associated with high Aix. • OCP’s may promote the development of hypertension by increasing Aix, possibly by suppressing endogenous estradiol.
[27]	The association of long-term outcome and biological sex in patients with acute heart failure from different geographic regions	Sex/gender and health outcomes: Cardiovascular diseases and related disorders	• Women with AHF have a lower 1-year mortality compared to men. • Women were less likely to receive evidenced-based treatment compared to men.
[28]	Sex differences in cardiometabolic disorders	Sex/gender and health outcomes: Cardiovascular diseases and related disorders	• Reviews clinical data regarding sex differences in cardiometabolic risk factors, pre-clinical cardiometabolic disease and overt cardiometabolic disease. • Reviews molecular mechanisms of sex differences in cardiometabolic disorders. • Advocates for developing gender-specific diagnostic tests and procedures. • Advocates for continued research on sex differences in heart disease and on sex-specific therapeutic interventions.
[29]	The case for sex- and gender-specific medicine	Sex/gender and health outcomes: Cardiovascular diseases and related disorders	• Reviews clinical evidence on sex differences in cardiovascular disease. • Advocates for sex/gender specific approach in diagnosis and treatment of cardiovascular disease.
[30]	Gender aspects suggestive of gastroparesis in patients with diabetes mellitus: A cross-sectional survey	Sex/gender and health outcomes: Diabetes mellitus	• Case control study suggesting that gastroparesis symptoms are more prevalent and more sever in female patients with DM. • Female patients had higher BMI and HgA1C. • Prevalence and severity of gastroparesis symptoms was higher in obese females with long standing, poorly controlled T2DM.
[31]	Vitamin B12 Deficiency and the Role of Gender: A Cross-Sectional Study of a Large Cohort	Sex/gender and health outcomes: B12 deficiency	• Cross sectional study in healthy individuals. • Prevalence of B12 deficiency was higher in men.
[32]	Gender as an independent risk factor for the components of metabolic syndrome among individuals within the normal range of body mass index	Sex/gender and health outcomes: Cardiovascular diseases and related disorders	• Cross sectional study in health individuals. • Male gender is an independent risk factor for all components of the Metabolic syndrome, apart from low HDL, which is more prevalent in women. • When comparing only post-menopausal women to men, differences become smaller.
[33]	Gender differences in the comprehension of care plans in an emergency department setting	Treatment decisions and care plans	• No gender-related differences were found in comprehension of care plan in the emergency department.
[34]	Is There Gender Discrimination in Acute Renal Colic Pain Management? A Retrospective Analysis in an Emergency Department Setting	Treatment decisions and care plans	• Men with renal colic had a higher VAS score and received more analgesics and opioids. • Non-Jewish women experienced longer waiting time until medical assessment compared to Jewish women.
[35]	The effect of childcare activities on cognitive status and depression in older adults: gender differences in a 4.4-year longitudinal study	Sex/gender and the human brain, cognition, and mood	• Daily childcare was associated with reduced rates of cognitive decline in elderly men and women. • Daily childcare was associated with decreased rates of depression in men but not in women. • Occasional childcare was associated with reduced depression rates in women and men.
[36]	Sex difference or hormonal difference in mental rotation? The influence of ovarian milieu	Sex/gender and the human brain, cognition, and mood	• Men perform better then female in the visuospatial Mental Rotation Test. (MRT) • After analyzing according to the menstrual cycle phase and OCP use, women with low- estradiol performed as good as men, and better than the high-estradiol group. • No gender differentiation was found in verbal memory control task but performance varied with hormonal milieu.
[37]	Crying, oral contraceptive use and the menstrual cycle	Sex/gender and the human brain, cognition, and mood	• Women in the reproductive reported feeling more like crying pre-menstrual but may not actually cry more during this phase. • Oral contraceptive use had no effect.
[38]	Yes, there is a female and a male brain: Morphology versus functionality	Sex/gender and the human brain, cognition, and mood	• Rejects the findings of a study that showed that brains of males and females are functionally and morphologically diverse and cannot fit into a male/ female category. • Claims that female and male brains are distinct. • Claims that testosterone has a crucial affect on the developing brain, and is responsible for sex differences in the brain.
[39]	Influence of gender, working field and psychosocial factors on the vulnerability for burnout in mental hospital staff: results of an Austrian cross-sectional study	Sex/gender and working conditions and promotion in healthcare	• Emotional exhaustion was higher in women working with patients compared to men working with patients. • Age above 45 was associated wit decreased burnout in men but not women. • There is a need for gender specific preventive strategies to reduce burnout.
[40]	Parenthood does not explain the gender difference in clinical position in academic medicine among Swedish, Dutch and Austrian physicians	Sex/gender and working conditions and promotion in healthcare	• Gender influences clinical position. • Female physicians publish fewer articles then male physicians. • Number of children or working hours did not explain gender differences in publication activity or clinical position. • Factors such as the academic working environment, may still disadvantage women’s progress.
[41]	Male Medical Students’ Gender-Role Conflict Is Associated with Their Discomfort With Dealing With Patients’ Sexual Health Issues	Sex/gender and medical education	• Knowledge about sexual health was associated with comfort. • Male students who had difficulty expressing affection towards men or expressing emotions were likely to feel uncomfortable asking patients about sexual health issues.
[42]	Integrating topics of sex and gender into medical curricula - Lessons from the international community	Sex/gender and medical education	• Advocates for inclusion of sex/gender difference in health and disease management into medical curricula. • Suggests methodologies and information resources.
[43]	Considering sex as a biological variable in preclinical research	Sex in preclinical research	• Sex is an important biologic variable and should be considered in pre-clinical research. • Suggests strategies to incorporate sex as a biological variable in study design, performance and analysis. • Suggests considering sex chromosomes and sex hormones in basic research and sex/gender aspects in animal studies.

In the following paragraphs, we discuss the reviewed medical and popular literature according to topics raised by feminist writings on sex/gender in health/medicine.

#### Is it possible to separate the effect of sex and gender on health?

Although it uses the word “gender”, GM focuses mostly on biological sex, stressing biological differences between the sexes in physiological and pathological conditions. However, this division ignores human complexity and the criticism of determinist models of sex differences highlighted by feminist thinkers since the 1990s [3] and subordinates the critical concept of gender to the biological concept of sex.

Many behavioral, psychological, and social variables correlate with sex category (being female or male). It is therefore often impossible to distinguish the contribution of these factors (i.e., gender) from that of biological variables (i.e., sex) to observed health differences between women and men. In addition, gender-related behaviors and experiences were shown to affect biological qualities thought to stem from sex category, such as levels of sex hormones, making the separation between sex effects and gender effects even more difficult [5, 44–47].

Indeed, many unacknowledged factors may mediate ostensibly sex-driven differences. For example, GM publications quote observational studies according to which women suffer from more cardiac sequalae after acute coronary syndrome (ACS) [17, 27]. However, a recent study demonstrated that gender roles, such as being the primary provider, employment, and household responsibilities, rather than sex, are those associated with prognosis after ACS [48]. Gender associated behaviors were shown to influence seemingly sex related differences in osteoporosis [49, 50] and melanoma [51, 52]. Researchers have shown that sex related differences documented in laboratory animals can stem from behavior and living condition and not from biological differences [46]. Thus, sex differences are often caused by other variables, that correlate with the sex category. Searching for these variables and their significance to health, instead of using sex as a proxy for their values, would benefit personalized medicine [6].

Feminist researchers pointed out that research often builds on a pre-assumption that sex differences in the brain exist [44] and that arguments about alleged sex differences that echo cultural stereotypes receive public attention [53]. It was shown that sex differences in the brain are often context- related, and change with time and circumstances [7, 47]. Of note, mothers were shown to behave differently towards male and female babies [54], implying that the brains of women and men are exposed to different stimuli from an extremely early stage of development.

While sex and gender are regarded as two separate entities [12], biological qualities of sex such as sex hormone levels are altered by gender related experiences and behaviors such as nurturing, competition and sexual activity in both men and women [55–57]. This suggests that social, material, and cultural factors likely contribute to some of the differences between men and women in health outcomes. It also suggests that addressing gender disparities is essential to improve health outcomes for women, and that both epidemiological and basic research should address the numerous social factors which differ between men and women. Gender disparities and their relation to health are addressed in a minority of the GM publication we reviewed [22, 28] and are mentioned in working field 2 of the EUGENMED report [12], but the vast majority of publications do not address gender issues. The EUGENMED workshop dedicated to basic research discusses biological sex alone, and does not acknowledge the data concerning the entanglement of biological sex and gender, nor does it call for research on this subject [12]. Moreover, some IGM officials explicitly state that “gender medicine is not feminist, it’s about real science” [58], thus denying the political and scientific origins of the GM project.

Exposure to physical and sexual violence in childhood and adulthood have a profound and prolonged impact on many women’s lives. Although violence is generally under-reported, the United Nations reported in 2012 that between ten and 40% of women worldwide experienced sexual violence during their lifetime and between seven and 68% experienced physical violence [59]. Studies have repeatedly shown the association of childhood abuse with cardiovascular [60, 61], autoimmune, metabolic diseases [62], chronic pain [63–65] and with mortality in women [66]. Studies discern long- lasting biological changes in abuse survivors such as increased pituitary stress response [67], increased inflammation [68] and even DNA changes such as decreased telomere length in leukocytes [69] and epigenetic changes in the brain, which can be transmitted to subsequent generations [70]. These gender-related life experiences often go unnoticed in the public sphere and in healthcare systems [71], and may mediate many seemingly sex differences in health.

Depression and anxiety are twice as common in women than in men. Abuse and violence increase the risk of depression, anxiety and post-traumatic stress disorder [72–74] mediated by chronic biological changes in multiple cellular and molecular components of brain function [75]. Failing to address the causative role of gender-related violence and discrimination in women’s mood disorders results in women being labeled “emotional” and “unstable”, bolstering discrimination and the silence surrounding gender-related violence. The GM studies and policies we reviewed refer to violence and childhood abuse only marginally, and do not address violence and abuse when discussing mood disorders [12]. The Israeli GM society, led by the former IGM president, states that trauma is less common in women on its webpage, reflecting a lack of understanding of the prevalence and consequences of childhood abuse and adult-life violence experienced by women [76]. Only one IGM member, Gillian Einstein, addresses violence in her scientific work [77] and public appearances.

Unfortunately, we do not fully understand the long-term health consequences of abuse and violence in women. Likewise, specific diagnostic and therapeutic interventions are not being developed. GM does not address these important issues, nor does it mention the urgent need to improve our understanding of the long-term health consequences of gender-related violence.

Some perceived sex differences in health may arise from diagnostic criteria that do not account for gender differences in manifestations of diseases. For example, depression in men may be overlooked when manifested as alcohol and substance abuse [78, 79]. Gender appropriate diagnostic criteria of osteoporosis improves its diagnosis and treatment in men [80]. Autism in women was shown to be underdiagnosed, probably because the tendency to internalize problems and camouflage social difficulties, as well as gender appropriate repetitive interests are common in autistic females [81]. These examples demonstrate that simply focusing on sex differences in epidemiology, without considering complex interactions with gender, can result in under-diagnosis and inappropriate treatment in both men and women.

#### Is there a binary division between the physiology of women and men?

Dividing men and women into two biological categories with different features and qualities constituted the basis for women’s oppression throughout history [82, 83]. Several GM publications assume the existence of biological differences between male and female brains, cognitive abilities, and emotional expressions, attributing these to biological factors such as sex hormones [36, 37]. However, scientific evidence shows that even when a statistically significant difference in found, considerable overlap in the distribution of measurements of single variables (e.g., specific psychological qualities and cognitive abilities) between the sexes exists [5, 6, 84]. For example, an extensive review of 26 meta-analyses looking for sex differences in psychological and cognitive traits found that, for almost all the traits studied, differences were close to zero or small and a considerable overlap existed [85].

In addition, when multiple variables are tested simultaneously in female and male brains, a mosaic distribution of “feminine” and “masculine” qualities across variables is found [7, 51, 86, 87]. This means that in an individual brain, each variable tested shows its own degree of similarity to the phenotype more common in females or in males, so that varying degrees of “femininity” and “masculinity” are found across variables in each person. Mosaic patterns were seen in brain structure on functional MRI, when assessing psychological traits by questionnaires [86], and even when assessing cellular brain structure postmortem [88]. Mosaic pattern are also seen in the effect of external stimuli, like stress, on brain function [89]. These important data shed light on the complex interactions between biological sex, the environment, and the brain, and highlight the fact that it is impossible to categorize human brains as ‘male’ or ‘female’. Of note, the groundbreaking study that delineated the brain mosaic theory was firmly rejected by the former IGM president [38].

#### Listening and learning from other disciplines and from women themselves

The women’s health movement empowered women to learn and share their health-related knowledge. The revolutionary book ‘Our Bodies Ourselves’, written by women for women, cherished women’s experience and challenged the authoritative position of the healthcare system. This enabled women to expose misconceptions and prejudice in medical practice. GM is practiced and discussed by physicians and scientists. In our review of GM work we did not find studies regarding women’s concerns in health, not a call for such work. While GM focuses mainly on cardiovascular health and diabetes [12, 18, 22, 26–28, 30], it is plausible that women from diverse backgrounds have different health concerns and priorities. The EUGENMED project involved patient’s organizations, but not feminist organizations, as stakeholders [12]. Empowerment of women regarding their health is also absent from GM discussions and recommendations.

#### Intersections between gender, oppression, and racial discrimination

Poverty, discrimination, economic insecurity and ethnic conflicts profoundly affect the epidemiology of common diseases and treatment outcomes [48]. These adversities generate chronic stress and affect nutrition, physical activity, exposure to pollution, access to healthcare and more. The capitalistic system generates and broadens economic inequalities between countries worldwide and within states and societies. “Black feminism” and intersectionality theory demonstrate how race, class, ability, and appearance interact with gender to generate privilege and discrimination [90]. GM publications recognize the effect of poverty and racial discrimination on cardiovascular risk [12, 28], however a call to improve and study minority women’s health is lacking. Minority women in the US, Canada, Israel, Europe, and Australia report discrimination within healthcare systems and discriminatory institutional policies and stigmas, with negative effects on their health [91, 92]. Gender discrimination in healthcare is suggested by the findings described in several of the studies we reviewed [16, 18–20, 23, 27]. For example – a study found that women undergoing hemodialysis in Austria were less likely to be treated via a vascular shunt and less likely to be referred to kidney transplantation [16]. Other studies showed that women with type 1 diabetes [20] and women hospitalized for heart failure [27] were less likely to be treated per current guidelines, that women were at higher risk for acute ischemic events in a cohort of patients after cardiac catheterization [19], and that women with type 1 diabetes were more likely to suffer hypoglycemia and severe hypoglycemia when treated in clinical trials of galgarin insulin [18]. Discrimination in healthcare practices and access to medical and social services may contribute to these and other [23] findings, however only 1 article [27] mentions this possibility. A discussion regarding the need for further studies looking specifically at discriminatory practices is also lacking. Racial discrimination in healthcare is not discussed at all in the publications we reviewed, and even refuted when faced with findings regarding inadequate treatment provided to Arab minority women in Israel [24].

## Discussion

The “Me-too” protest against sexual violence and the “Black Lives Matter” movement reminded us that gender related violence and racial discrimination are prevalent even among seemingly liberal institutions in western societies. These uprisings share values and practices with the feminist movement, empowering women and minorities and cherishing their voices and perspectives. They teach us that real change is accomplished only by questioning the practices, interests, and power-structures of institutions.

GM has brought the issue of sex/gender and general health to the forefront of popular and professional discourse, appropriating, and mainstreaming the discussion that was initiated by the feminist women’s health movement in the 1960s. This process has obvious advantages and opportunities, such as raising awareness of health professionals, institutions, and regulatory agencies to gender differences in health, allocation of funds to research on gender and health, and better designed pharmaceutical studies. However, this mainstreaming has been accompanied by the return of professional dominance, while the voices of feminist activists go unheard. Moreover, GM ignores important scientific progress, made by feminist scientists, regarding the complex associations between sex, gender, and health. By stressing the biological division between sexes, on the one hand, and under-representing the toll of violence, oppression, ethnic conflicts, and discrimination on the lives and health of women, on the other, GM accepts conservative positions on sex and gender and reaffirms the current practices of healthcare systems worldwide. Generally, it does not posit poignant criticism to mainstream medicine, and the topics studied tend to avoid more contested health issues such as chronic pain syndromes, sexual abuse, ethnic conflict, the health consequences of beauty standards, and others.

### A way forward

Feminist scientists have shown that much can be achieved by studying the mechanisms linking biology, gender, and society. A continued effort in this direction is required to improve our understanding of these mechanisms, and to implement this knowledge into clinical practice. An approach that integrates feminist epistemology and methodology into the study and practice of medicine and strives to understand the complexity of gender can improve the health of both women and men [79, 80] worldwide. Feminist activists should work together with physicians to re- define “Gender Medicine”, prioritize research and policy topics, and participate in the design of clinical studies. Efforts should be made to listen to diverse women, learn about the health challenges they face and incorporate their priorities into policies and studies. Studies that critically examine healthcare systems and the bio-scientific world for discriminatory practices and blind spots, and studies that examine the health toll of diverse forms of gender related violence and oppression should be encouraged.

## Conclusion

Our review of the IGM/ ISGSCM indicate that while their work focuses on sex differences, it neglects the influence of gender, namely the social aspect of being a woman or a man, on biology, physiology, and health. We found that for the most part, their writing ignores the effect of gender norms, gender-related behaviors, and gender-related violence on biology and health. Moreover, it endorses a binary vision of 2 distinct sexes with different biological qualities, while overlooking the evidence that indicate a more complex social and biological reality. Indeed, the IGM/ ISGSCM work may improve some aspects of women’s health, however we should aim to promote a wider approach to gender and medicine – one that studies complex interactions between society and biology and that tackles difficult subjects such as debilitating chronic pain syndromes, violence, and health concerns of racial minorities. We believe that integrating the achievements of the IGM, those of the feminist women’s health movement and of current feminist scientists and activists can bring about a deep and meaningful change in the health of women worldwide.

## Data Availability

N/A
